# Integrating a Positron Emission Tomography/Computed Tomography Into the National Health System of Cyprus: Will It Return on Its Investment?

**DOI:** 10.3389/fpubh.2021.607761

**Published:** 2021-03-10

**Authors:** Dimitra Kefallonitou, Irene Polycarpou, Kyriakos Souliotis, Konstantinos Giannakou

**Affiliations:** ^1^Department of Health Sciences, School of Sciences, European University Cyprus, Nicosia, Cyprus; ^2^Faculty of Social and Political Sciences, University of Peloponnese, Corinth, Greece; ^3^Health Policy Institute, Maroussi, Greece

**Keywords:** positron emission tomography-computed tomography, PET/CT, Cyprus, economic feasibility study, cost analysis

## Abstract

A European Union (EU) member state, Cyprus is a country with a population of ~850,000 citizens. According to the Cyprus Ministry of Health, since 2009, more than 3,000 new incidents with neoplasm are diagnosed every year (i.e., 3% increasing rate). Projections estimate an average annual increase of 2.2% of new incidents until 2040. However, the National Health System (NHS) of Cyprus lacks a Positron Emission Tomography/Computed Tomography (PET/CT) care framework and infrastructure. Patients can only have a PET/CT exam in the private sector, either in Cyprus or a neighboring country (e.g., Greece or Israel). This requires the government of Cyprus to cover financial expenses related to medical treatments while the patients may also need to cover their expenses for traveling to a neighboring country. This study presents a cost analysis to examine whether the integration of a PET/CT with, or without, an F^18^-FDG cyclotron unit in the NHS of Cyprus is an efficient investment that can be recovered within the unit's service life. To perform this study, we estimated necessary resources for purchasing and operating such unit for a period of 15 years. The results of this study indicate that an investment in a PET/CT unit is not financially viable. Alternatives, such as the reimbursement of PET/CT operated by the private sector is recommended.

## Introduction

Health systems aim to provide a comprehensive range of services to the entire population of a country and ensure equitable access to quality services. One of the main objectives of health systems is the improvement of the population's health and the enhancement of patient experience (1, 2). In the context of evolving pressures and growing demand, the administrators face the challenge of ensuring the sustainability of a high performing health system.

Health system capacity planning is important as it aims to create the conditions for the development of a truly high performing system (3, 4). The responsibility for developing the overall framework for financing and organizing a health care system usually lies with the central government whilst in most countries, health capacity planning takes place at national, regional, or local level. The administration of a health system is often shared by central and regional authorities (5). Key strategy elements in matters of politics, constitutes the introduction of reinforcement tools, such as Health Technology Assessment (HTA), aiming to inform technology-related policy making in health care. The assessment of medical devices and equipment, improves procedures and could also assist to derive important information regarding efficacy, quality, appropriateness, cost–effectiveness and efficiency dimensions of such technologies, determining the conditions for which these health interventions/technologies should be adopted by the health system (6).

Diagnostic equipment, such as the Positron Emission Tomography/Computed Tomography (PET/CT), comprise an important category of a health system capacity. PET/CT units are expensive and therefore operating policies are usually oriented toward achieving universal utilization (7). To avoid excess capacity or unnecessary usage, procurement of PET/CT units must be regulated by the health systems with specific processes. In particular, the planning of hospital capacity involves several dimensions including capital investment in existing facilities and new developments, investment in expensive equipment and technology, service delivery, and allocation of human and financial resources (5).

Cyprus is a small island country located in the Mediterranean Sea, is classified as a developed economy and one that faces similar challenges, such as an aging population and an increase in non-communicable diseases (e.g., cancer, cardiovascular, etc.) with countries of similar size and population (8, 9). Data from the Ministry of Health of the Republic of Cyprus, shows a steady annual increase of 3% in the number of new incidents that have been diagnosed with neoplasm every year since 1998 which now exceed 3,000 per annum (10).

The Ministry of Health of the Republic of Cyprus with the assistance of the European Commission is responsible for the development of strategic and operational blueprints to support planning and implementation of new technologies in the field of health (capacity planning). This can cover issues pertaining health professionals and infrastructure in both the public and the private health sector. This planning process is essential toward ensuring the proper organization and operation of health systems, under the constraints of financial viability and the provision of quality health services. It also helps in optimizing throughput across different areas of health services, to avoid creating shortages or oversupply in various areas of health services. Currently, Cyprus does not have a formalized HTA process for the assessment of medical devices (11). Other countries of similar population size (~850,000 citizens), such as Luxembourg use HTA to inform the development of quality standards with regards to medical devices and/or other technologies. Likewise, Latvia uses HTA to inform pricing decisions for medical devices, while Malta indicated not having a formalized HTA process for medical devices (11).

Cyprus has undergone a major transformation to establish a new National Healthcare System (NHS). It aims to provide universal coverage to its population through the merging of public and private sector, improve accessibility and address inefficiencies in service delivery (12). The NHS of Cyprus is designed with the objective of offering adequate, by European Union standards, health services. However, a PET/CT unit is not currently available in the NHS of Cyprus to support oncologic incidents. Therefore, patients are granted financial support from the government of Cyprus (i.e., Ministry of Health) to perform a PET/CT in the private sector or in a neighboring country, such as Greece or Israel. The present study aims to examine whether an investment by the Cyprus NHS of a PET/CT unit can be financially sustainable without taking into consideration other essential factors, such as the value of extending patients' life expectancy.

## Materials and Methods

This study is a cost analysis of revenues and expenses for operating a departmental PET/CT that uses F^18^-FDG radiopharmaceutical. A detailed estimate of the unit's operational expenses (PET/CT unit supplies, overheads, salaries) and revenues has been projected for a 15-year period. The abovementioned procedure has been conducted following the same principles as the ones used in a previous study (13). In this study, a detailed estimation of expenses (unit purchasing and annual service, hospital's overheads, e.g., heating electricity and water supply as well as security or facility management supportive services, salaries, radiopharmaceutical cost, and other examination costs) and revenues (mainly remuneration by the National Organization for the Provision of Health Services) of the PET/CT unit operation for a 15 year period were developed. The referenced study concluded in the base case scenario that the integration of the PET/CT unit by the NHS is not financially viable and therefore two alternatives scenarios are also examined, where in scenario A the intention is to increase the number of served incidents, and scenario B considers the combined purchase of the PET/CT unit with a separate cyclotron unit.

To perform such an analysis, information from different sources has been gathered in line with the World Health Organization (WHO) guidance (14). According to this, it is essential to map in detail the local geographical and public health conditions (e.g., epidemiology), the actual service delivery situation (e.g., availability and accessibility), the medical device situation (including status and condition), the required human resources (availability, capacity and capability) as well as the financial situation (available budget) for analyzing and defining appraisals. For each year considered, detailed analysis of revenues and expenses was performed. Also, all important factors which relate to the present usage of this technology due to oncological incidents, as well as the projections were defined. According to recorded statistics by the Ministry of Health of the Republic of Cyprus, the oncologic incidents have increased by 130.7% among males, 100.1% among females, resulting in an aggregate increase of 114.8% across the entire population between 1998 and 2014 (Supplementary Figure 1) (10). The above data are corroborated by the published information of the Bank of Cyprus Oncology Centre whereby a similar trend is recorded (Supplementary Table 1) (15).

Information regarding the number of patients examined using the PET/CT available in the private sector of Cyprus and for those who received financial support by the NHS to be examined abroad were retrieved from the General Director of the Ministry of Health (16). During 2018, 205 patients had a PET/CT scan in the private sector while another 73 patients have received financial aid by the Cyprus NHS to have the PET/CT scan in neighboring countries, such as Greece and Israel (13). The remuneration of the NHS to support patients for both choices is set at €1,150 per examination. According to WHO's Global Cancer Observatory (17), it is expected that the growth of incidents in Cyprus will increase by 38.1% over the next 22 years (2018–2040), which translates to an annualized growth rate of 2.2% (Supplementary Table 2).

The cost for supplying the unit with the required ^18^F-FDG radiopharmaceutical from Greece is expected to reflect a price of €700 per examination relying on the Price Observatory of the Greek Ministry of Health (18). Increasing the number of examinations every year can increase the negotiating position of the NHS of Cyprus for discounted prices on consumable, potentially leading to an annual reduction of 1% per unit.

It is essential to point out that the total cost of purchasing and installing a new PET/CT unit capable to support the needs of the NHS of Cyprus is estimated at €2.5 million, including the PET/CT unit purchase, installation and construction works. An expected cost for complementary supporting annual services for such a unit is €120,000.

To determine the labor cost for operation of the unit, we assumed that the following staff are required: one doctor, one medical-physicist, one radiographer, one nurse, and one secretary, all of them with an average of 15 years relevant work experience. These professionals will be remunerated according to the payroll cited by the Treasury of the Republic of Cyprus and adjusting for annual salary increases in line with inflation (1% per year) (19). To perform a cost analysis, a 15-year period is considered reasonable for determining the sustainability of such a decision, as this is considered to be the average Service Life limit of such unit. The integration of a PET/CT technology to the NHS system is expected to produce a further increase of 5% in the usage of this technology by oncologists. For this purpose, the incidents which were reported in 2018 by the Ministry of Health, are expected to increase by the compound effect of the projections of the Global Cancel Observatory and the integration of the technology to the NHS of Cyprus. As a result, growth in the usage of such technology in the upcoming years is presented in Supplementary Table 3.

On an annual basis, staff involved in health activities typically work 220 days a year. Assuming that the entire team dedicates 40% of their work effort, around 2 working days per week, for the operation of a PET/CT unit, the number of daily patients served is expected to triple by 2035 reaching 10.56 patients on daily basis. It is essential to assume that such a decision is taken in the coming months and that a PET/CT unit starts to operate properly by 1st January 2021. Even if there is a necessity to estimate a cost for the facilities that will accommodate the unit, for simplification purposes it is considered that space is already available in the institution that supports centrally the NHS of Cyprus for oncological incidents and that any cost required is included in the €2.5 million mentioned before.

For each examination, some medical consumables must be foreseen for the execution of the scan, such as venipuncture, syringe, oral contrast, and blood sugar test, with a cost of €25 per examination. Finally, it has been estimated that the overheads for an examination is estimated to ~€80 per examination by taking into consideration hospital's handling services, such as security and cleaning, secondary device purchase, operation, and renewal (computers and printings), building renovations, utility bills for heating, cooling, lighting, telecommunications, etc. Since the results of the above-mentioned estimation was not financially viable, two alternative scenarios were examined. The variables used for each scenario are reported in Supplementary Table 4.

## Results

Table 1 provides an overview of the projection for the evaluation of the feasibility study, showing a cumulative operating loss of 2.2 million € by 2035. Such a figure significantly deviates from a break-even scenario due to the limited number of patients served on annual basis, translated to max 4.2 patients served on daily basis by 2035, even if the losses per examination are significantly reduced from €550 in 2021 to €87 in 2035.

**Table 1:** Economic feasibility estimation based on projections.

Year	2021	2022	2023	2024	2025	2026	2027	2028	2029	2030	2031	2032	2033	2034	2035
Incidents	347	373	401	431	463	498	534	573	615	660	707	758	812	869	930
**Costs per examination**															
Overheads	80	80	80	80	80	80	80	80	80	80	80	80	80	80	80
Salaries	270	253	238	223	210	197	186	175	165	155	146	138	130	123	116
Purchasing	279	279	279	279	279	279	279	279	279	279	279	279	279	279	279
Services	346	322	299	278	259	241	225	209	195	182	170	158	148	138	129
Consumables	25	25	25	25	25	25	25	25	25	25	25	25	25	25	25
^18^F-FDG	700	693	686	679	672	666	659	652	646	639	633	627	620	614	608
Total	1,700	1,652	1,607	1,565	1,525	1,488	1,453	1,420	1,389	1,360	1,332	1,306	1,282	1,259	1,237
**Annual results**															
Costs	589,227	616,081	644,417	674,625	706,501	740,641	776,494	814,319	854,508	897,308	942,465	989,991	1,040,458	1,093,449	1,149,574
Revenues	398,690	428,965	461,190	495,818	532,668	572,428	614,539	659,327	707,274	758,707	813,396	871,407	933,448	999,102	1,069,131
	−190,538	−187,116	−183,227	−178,807	−173,834	−168,214	−161,955	−154,991	−147,234	−138,601	−129,069	−118,584	−107,010	−94,348	−80,442
**Cashflow (end year)**															
Cumulative	−190,538	−377,654	−560,881	−739,688	−913,522	−1,081,736	−1,243,691	−1,398,682	−1,545,916	−1,684,518	−1,813,587	−1,932,170	−2,039,181	−2,133,528	−2,213,971

Table 2 presents the alternative scenario A, where the intention is to increase the number of incidents to reach a neutral cash flow at the end of 2035. Increasing the number of incidents will impact several parameters (e.g., the purchasing cost per examination will be reduced proportionally from €279 to €121). The only parameter that increases comparing with the estimation that is based on projection, is the labor cost due to the increased human resource capacity with 5 days a week engagement for the involved staff in the PET/CT unit and 50% for the director of the department. Obviously out of such attempt, several unit cost values of the table are adapted because they are sensitive to the number of examinations.

**Table 2:** Economic feasibility estimation according to alternative scenario A.

Year	2021	2022	2023	2024	2025	2026	2027	2028	2029	2030	2031	2032	2033	2034	2035
Incidents	1,375	1,375	1,375	1,375	1,375	1,375	1,375	1,375	1,375	1,375	1,375	1,375	1,375	1,375	1,375
**Costs per examination**															
Overheads	80	80	80	80	80	80	80	80	80	80	80	80	80	80	80
Salaries	170	172	173	175	177	179	181	182	184	186	188	190	192	194	195
Purchasing	121	121	121	121	121	121	121	121	121	121	121	121	121	121	121
Services	87	87	87	87	87	87	87	87	87	87	87	87	87	87	87
Consumables	25	25	25	25	25	25	25	25	25	25	25	25	25	25	25
^18^F-FDG	700	693	686	679	672	666	659	652	646	639	633	627	620	614	608
Total	1,184	1,178	1,173	1,168	1,163	1,158	1,153	1,148	1,144	1,139	1,134	1,130	1,126	1,121	1,117
**Annual results**															
Costs	1,627,357	1,620,070	1,612,903	1,605,854	1,598,924	1,592,112	1,585,416	1,578,836	1,572,372	1,566,022	1,559,787	1,553,665	1,547,656	1,541,759	1,535,974
Revenues	1,581,250	1,581,250	1,581,250	1,581,250	1,581,250	1,581,250	1,581,250	1,581,250	1,581,250	1,581,250	1,581,250	1,581,250	1,581,250	1,581,250	1,581,250
	−46,107	−38,820	−31,653	−24,604	−17,674	−10,862	−41,66	2,414	8,878	15,228	21,463	27,585	33,594	39,491	45,276
**Cashflow (end year)**															
Cumulative	−46,107	−84,926	−116,579	−141,183	−158,858	−169,719	−173,885	−171,471	−162,592	−147,364	−125,901	−98,316	−64,721	−25,230	20,046

Table 3 shows the alternative scenario B, which combines the purchase of the PET/CT unit, a separate ^18^F-FDG cyclotron unit of about 2.5 million € (purchasing cost), that will most likely reduce the cost that relates to the supply of the radiopharmaceutical to €200 per examination (20). Comparing with the estimation that is based on projection (Table 1), the only input parameter that varies is the cost of the radiopharmaceutical. Scenarios' cash flow results to a positive cumulative cashflow of 1.7 million € by 2035. Such an amount covers 68% of the purchasing costs for integrating the complementary equipment (cyclotron) by any other public non-profit structure (e.g., structure serving the NHS, research center, university) and as a result contributes to the operational independence of the NHS from external suppliers.

**Table 3:** Economic feasibility estimation according to alternative scenario B.

Year	2021	2022	2023	2024	2025	2026	2027	2028	2029	2030	2031	2032	2033	2034	2035
Incidents	347	373	401	431	463	498	534	573	615	660	707	758	812	869	930
**Costs per examination**															
Overheads	80	80	80	80	80	80	80	80	80	80	80	80	80	80	80
Salaries	270	253	238	223	210	197	186	175	165	155	146	138	130	123	116
Purchasing	279	279	279	279	279	279	279	279	279	279	279	279	279	279	279
Services	346	322	299	278	259	241	225	209	195	182	170	158	148	138	129
Consumables	25	25	25	25	25	25	25	25	25	25	25	25	25	25	25
^18^F-FDG	200	200	200	200	200	200	200	200	200	200	200	200	200	200	200
Total	1,200	1,159	1,121	1,086	1,053	1,022	994	968	943	921	899	880	861	844	828
**Annual results**															
Costs	415,884	432,185	449,486	468,016	487,683	508,837	531,193	554,919	580,257	607,375	636,156	666,633	699,166	733,543	770,151
Revenues	398,690	428,965	461,190	495,818	532,668	572,428	614,539	659,327	707,274	758,707	813,396	871,407	933,448	999,102	1,069,131
	−17,194	−3,221	11,704	27,802	44,985	63,591	83,345	104,408	127,017	151,332	177,240	204,774	234,282	265,559	298,980
**Cashflow (end year)**															
Cumulative	−17,194	−20,415	−8,711	19,091	64,076	127,667	211,012	315,420	442,437	593,769	771,009	975,783	1,210,065	1,475,624	1,774,604

## Discussion

In this study, a cost analysis was performed to estimate whether the integration of a PET/CT unit for oncological patients by the NHS of Cyprus is a good return on investment. Our estimations show that an investment of a sole PET/CT unit is not viable. Considering the expenses that the NHS of Cyprus is currently reimbursing for the PET/CT exam in the private sector on an annual basis, such investment is not sustainable in absolute economic terms.

To increase the operable hours of the unit and therefore reduce costs to make the investment viable, it is necessary to find a significant number of patients. Usually, the strategy of a public healthcare system is not to maximize patient flow, but to serve its patients that are typically long-term insured. Respectively, Germany is a typical example of a Long-Term Care Insurance Program where all citizens are covered (21). It is worth mentioning that the General Healthcare System (GHS) of Cyprus is a modern, patient-centric healthcare system with the aim of delivering quality healthcare services to its beneficiaries (22).

An alternative scenario that was examined in our analysis, was to combine the investment of PET/CT scan with a complementary parallel investment by the NHS of Cyprus on a cyclotron unit. Using this approach, the cost for supplying the mandatorily required ^18^F-FDG radiopharmaceutical, generates more value (€500 in savings per exam) that could be used by the NHS of Cyprus for recovering this additional investment. In this scenario by 2035, an amount of around €1.7 million will constitute additional savings which is not sufficient, given that a cyclotron unit capable to serve a PET/CT unit requires ~€2 million.

A cost analysis of PET/CT has not been widely performed and there are doubts whether countries that do not have sufficient resources should invest in this system (23). Eurostat examined the availability of PET/CT scanners among the EU Member States. The PET/CT units which are available per 100.000 inhabitants within the Union Market is between 0.05 and 0.7 with an average of 0.19. Considering that in Cyprus this number is 0.12 per 100.000 inhabitants we can assume that the already available PET/CT unit belonging to the private sector, serves sufficiently the needs of such medical technology/imaging equipment (24). Examining Member States of the European Union with similar population, Luxembourg (0.2 per 100,000 inhabitants) and Latvia (0.1 per 100,000 inhabitants) have one PET/CT unit each, and Malta has two PET/CT units (0.4 per 100,000 inhabitants) (24, 25).

A previous study examined the cost of providing PET/CT services in an Indian hospital. The average number of daily PET/CT scans was 30 whilst the annual cost of providing PET/CT scan services was estimated to be 65,311,719 INR (Indian Rupees) (USD1,020,496) (26). The unit cost of PET/CT scan was estimated at 9,625.92 INR (USD150 ~ €133), while in the case of Cyprus the examination costs are at least 5 times higher regardless of the initial scenario and the average number of daily PET/CT scans should be at least 15 scans in a normal working day (13), in order to make the investment financially viable.

A previous cost analysis of Berger et al. of six major hospitals and two academic medical centers in the United States, three hospitals manufacture the radiopharmaceutical in their premises, also other three purchase the radiopharmaceutical and two of them do both (27). The total mean cost per scan using manufactured ^18^F-FDG was $1,885 (around €1,680) and using purchased ^18^F-FDG was $1,898 around €1,692). Similarly, in our study, one of the two alternative scenarios have examined the option to purchase cyclotron within the hospital, which in the case of Cyprus did not reach a breakeven point within a 15-year period. The installation of a PET/CT unit from the NHS of Cyprus could return its investment if the initiative would be financed or co-financed by third parties, such as EU funds or private sponsorship.

This study has some limitations. Initially, the cost for purchasing a PET/CT unit that will be installed in Cyprus is based on the cost that had been forecasted for Greece. Similar argumentation can be expressed in the second scenario for the purchase of a cyclotron unit. Furthermore, it is essential to underline that since the study focuses on PET/CT units, the values which relate to the cost for supplying the radiopharmaceutical are based either on the price the private sector is currently supplied or the results that have been presented in another scientific article. It is also essential to point out that the examinations forecast which will be executed can be questioned, even if the assumption according to which the annual demand that has been calculated is based on reasonable expectations. Finally, only the NHS currently provides the option to its patients to have a PET/CT exam in private sector, focusing on social impact is not considered essential in this particular case. Despite these caveats, the present study would be useful for policymakers and public health officials of other countries with similar characteristics with Cyprus.

## Conclusion

To the best of our knowledge, this is the first cost study that examines the economic feasibility of integrating a PET/CT unit in the NHS of Cyprus. Based on this study, from a cost viewpoint, investing in a PET/CT unit by the country is not financially viable, and as a result the write off period will not lie within the reference service life of the unit. Alternatives, such as reimbursement of PET/CT owned/operated by the private sector are recommended. The present study may be useful for examining the integration of expensive healthcare equipment (e.g., PET/MRI) in small sized countries, such as Cyprus.

## Data Availability Statement

The original contributions presented in the study are included in the article/Supplementary Material, further inquiries can be directed to the corresponding author/s.

## Author Contributions

DK conceived the study, conducted the analysis, and wrote the first draft. IP wrote and interpreted the results. KS supervised the study, supported the analysis, and contributed in the interpretation of results. KG supervised the study, wrote the manuscript, and interpreted the results. All authors read and approved the final manuscript.

## Conflict of Interest

The authors declare that the research was conducted in the absence of any commercial or financial relationships that could be construed as a potential conflict of interest.
