# Supplementary material for: Integrating a Positron Emission Tomography/Computed Tomography Into the National Health System of Cyprus: Will It Return on Its Investment?
Source: Front Public Health. 2021 Mar 10;9:607761. doi: 10.3389/fpubh.2021.607761 (PMC7987837; doi:10.3389/fpubh.2021.607761)
Supplement: Supplementary file 1 [file Table_1.DOCX]

**Supplementary Figure 1:** Number of oncologic incidents per year over the period 1998 – 2014 by sex (1)

1. Demetriou A. Αρχείο Καρκίνου Κύπρου Στατιστικά Στοιχεία 2016 [Available from: <https://www.moh.gov.cy/Moh/MOH.nsf/All/9245B3902339397CC22579C60026560F/$file/Cyprus%20Cancer%20Registry%20-%20Summary%20Results%201998-2016%20.pdf>.]
