# Supplementary material for: Integrating a Positron Emission Tomography/Computed Tomography Into the National Health System of Cyprus: Will It Return on Its Investment?
Source: Front Public Health. 2021 Mar 10;9:607761. doi: 10.3389/fpubh.2021.607761 (PMC7987837; doi:10.3389/fpubh.2021.607761)
Supplement: Supplementary file 2 [file Table_2.DOCX]

**Supplementary Table 1:** Supported oncologic incidence by Bank of Cyprus Oncology Centre(1)

| **Year** | 2006 | 2007 | 2008 | 2009 | 2010 | 2011 | 2012 | 2013 | 2014 | 2015 | 2016 | 2017 | 2018 |
| --- | --- | --- | --- | --- | --- | --- | --- | --- | --- | --- | --- | --- | --- |
| **Incidence** | 1586 | 1769 | 1800 | 1858 | 2024 | 2066 | 2116 | 2108 | 2255 | 2492 | 2657 | 2506 | 2308 |

1. (BOCOC) BoCOC. ΑΠΟΛΟΓΙΣΜΟΣ 2018 2019 [Available from: <http://www.bococ.org.cy/sites/default/files/inline-files/Annual_Report_2018.pdf>.]
