# Supplementary material for: Integrating a Positron Emission Tomography/Computed Tomography Into the National Health System of Cyprus: Will It Return on Its Investment?
Source: Front Public Health. 2021 Mar 10;9:607761. doi: 10.3389/fpubh.2021.607761 (PMC7987837; doi:10.3389/fpubh.2021.607761)
Supplement: Supplementary file 3 [file Table_3.DOCX]

**Supplementary Table 2:** Projections of incidents growth until 2040 (WHO-Global Cancer Observatory)(1)

| **Year** | **Incidents** | **Incidents growth basis year 2018** | **Year by year growth** |
| --- | --- | --- | --- |
| 2018 | 4829 |  |  |
| 2019 | 4956 | 2.6% | 2.6% |
| 2020 | 5083 | 5.0% | 2.5% |
| 2021 | 5212 | 7.3% | 2.5% |
| 2022 | 5344 | 9.6% | 2.5% |
| 2023 | 5475 | 11.8% | 2.4% |
| 2024 | 5609 | 13.9% | 2.4% |
| 2025 | 5742 | 15.9% | 2.3% |
| 2026 | 5880 | 17.9% | 2.3% |
| 2027 | 6015 | 19.7% | 2.2% |
| 2028 | 6149 | 21.5% | 2.2% |
| 2029 | 6285 | 23.2% | 2.2% |
| 2030 | 6424 | 24.8% | 2.2% |
| 2031 | 6562 | 26.4% | 2.1% |
| 2032 | 6698 | 27.9% | 2.0% |
| 2033 | 6836 | 29.4% | 2.0% |
| 2034 | 6971 | 30.7% | 1.9% |
| 2035 | 7107 | 32.1% | 1.9% |
| 2036 | 7250 | 33.4% | 2.0% |
| 2037 | 7384 | 34.6% | 1.8% |
| 2038 | 7525 | 35.8% | 1.9% |
| 2039 | 7666 | 37.0% | 1.8% |
| 2040 | 7803 | 38.1% | 1.8% |
|  |  | **Average annual:** | **2.2%** |

1. WHO. Global Cancer Observatory: Cancer Tommorow - Estimated number of incident cases from 2018 to 2040, all cancers, both sexes, all ages 2020 [Available from: <https://gco.iarc.fr/tomorrow/graphic-isotype?type=0&type_sex=0&mode=population&sex=0&populations=196&cancers=39&age_group=value&apc_male=0&apc_female=0&single_unit=500000&print=0>.]
