# Supplementary material for: Integrating a Positron Emission Tomography/Computed Tomography Into the National Health System of Cyprus: Will It Return on Its Investment?
Source: Front Public Health. 2021 Mar 10;9:607761. doi: 10.3389/fpubh.2021.607761 (PMC7987837; doi:10.3389/fpubh.2021.607761)
Supplement: Supplementary file 4 [file Table_4.DOCX]

**Supplementary Table 3:** Projections of PET/CT examinations until 2035

| **Year** | **Global Cancer Observatory** | **Global Cancer Observatory +5%** | **Incidents** |
| --- | --- | --- | --- |
| **2018** |  |  | 278 |
| **2019** | 2.6% | 103.1% | 299 |
| **2020** | 2.5% | 103.0% | 322 |
| **2021** | 2.5% | 103.0% | 347 |
| **2022** | 2.5% | 103.0% | 373 |
| **2023** | 2.4% | 102.9% | 401 |
| **2024** | 2.4% | 102.9% | 431 |
| **2025** | 2.3% | 102.8% | 463 |
| **2026** | 2.3% | 102.9% | 498 |
| **2027** | 2.2% | 102.8% | 534 |
| **2028** | 2.2% | 102.7% | 573 |
| **2029** | 2.2% | 102.7% | 615 |
| **2030** | 2.2% | 102.7% | 660 |
| **2031** | 2.1% | 102.6% | 707 |
| **2032** | 2.0% | 102.5% | 758 |
| **2033** | 2.0% | 102.5% | 812 |
| **2034** | 1.9% | 102.4% | 869 |
| **2035** | 1.9% | 102.4% | 930 |
