# Supplementary material for: Integrating a Positron Emission Tomography/Computed Tomography Into the National Health System of Cyprus: Will It Return on Its Investment?
Source: Front Public Health. 2021 Mar 10;9:607761. doi: 10.3389/fpubh.2021.607761 (PMC7987837; doi:10.3389/fpubh.2021.607761)
Supplement: Supplementary file 5 [file Table_5.DOCX]

**Supplementary Table 4:** Assumptions for projections and alternative scenarios

|  | **Projections** | **Alternative scenario A** | **Alternative scenario B** |
| --- | --- | --- | --- |
| **Incidents** | Based on projections | Introduce incoming patients for examination | Based on projections |
| **Overheads** | € 80/examination | | |
| **Workforce weekly engagement** | 20% for:  - unit’s director  40% for:  - one doctor  - one radiophysician  - one radiologist  - one nurse  - one secretary | 50% for:  - unit’s director  100% for:  - one doctor  - one radiophysician  - one radiologist  - one nurse  - one secretary | 20% for:  - unit’s director  40% for:  - one doctor  - one radiophysician  - one radiologist  - one nurse  - one secretary |
| **PET/CT Purchase** | € 2.5 million | | |
| **Cyclotron Purchase** | No investment by the NHS | | € 2.5 million (**not** included in the calculations) |
| **Annual service** | € 120 thousand | | |
| **Consumables** | € 25/examination | | |
| **^18^F-FDG** | € 700/examination deducted every year by 1% | € 700/examination deducted every year by 1% | € 200/examination |
